# Perceived barriers and enablers of physical activity in postpartum women: a qualitative approach

**DOI:** 10.1186/s12884-016-0908-x

**Published:** 2016-06-02

**Authors:** Maryam Saligheh, Beverley McNamara, Rosanna Rooney

**Affiliations:** School of Occupational Therapy and Social Work, Faculty of Health Sciences, Bentley Campus, Curtin University, Perth, WA Australia; School of Psychology and Speech Pathology, Faculty of Health Sciences, Bentley Campus, Curtin University, Perth, WA Australia; Discipline of Exercise and Sport Science, Faculty of Health Sciences, Cumberland Campus, University of Sydney, 75 East St, Lidcombe, NSW Australia

**Keywords:** Postnatal women, Exercise, Barriers, Enablers

## Abstract

**Background:**

Postpartum women’s recovery from birth can be assisted through increased physical activity (PA). However, women face substantial barriers to participating in exercise and require support to enable them to benefit from increased PA.

**Methods:**

This study sought to explore women’s beliefs about and experiences of PA and exercise during the 6 weeks to 12 months postpartum period. A cohort of 14 postpartum women from a survey study of the barriers and enablers to exercise participation agreed to take part in interview sessions to provide an in-depth understanding of the women’s perceptions of the postpartum period and their physical activity during this time.

**Results:**

Findings are presented with reference to the social ecological framework and indicate postpartum women face substantial personal and environmental barriers to PA and exercise participation: fatigue, a lack of motivation and confidence, substantial time constraints, lack of access to affordable and appropriate activities and poor access to public transport. In contrast, enablers such as possessing greater social support, in particular partner support, improved PA and exercise participation.

**Conclusions:**

The findings encourage facilitation of exercise through mothers’ groups, mothers’ exercise clubs or postnatal classes suggesting behavioral and social change is needed. Interaction between individuals, community, organizations and policy makers is required. In addition, the provision of specifically tailored and appropriate exercise programs could potentially enable increased PA in postpartum women, thereby improving their health.

**Electronic supplementary material:**

The online version of this article (doi:10.1186/s12884-016-0908-x) contains supplementary material, which is available to authorized users.

## Background

Despite the positive health outcomes that are associated with PA [1, 2], studies show that 25 % of women in the United States [3] and 36 % in Australia [4] do not participate in regular PA. The PA participation trend is similar but even lower for women in the postpartum period [5, 6] who appear to devote most of their time to household chores and care-giving and little time to exercise or sporting activities [7]. Studies indicate that PA declines during pregnancy [8, 9] and may remain low throughout the postpartum period [10]. Women who maintain or increase their sport and exercise from pre-pregnancy through to the postpartum period experience better well-being compared to women who do not [6]. These findings are of concern as the physical and psychological benefits of PA are well documented [11, 12]. One study suggests that barriers to physical activity have a great impact on exercise participation outcomes in postpartum women [13], yet the specific barriers to PA are not well defined or understood and there is a lack of recent research, which may help clarify the issues and indicate solutions.

Barriers to PA can be divided into environmental and personal factors, and postpartum studies have suggested the importance of considering both factors prior to the implementation of any exercise program [1, 8]. Personal factors are those related to the mothers’ own circumstances, such as income and the number of children under her care [5, 9, 14, 15]. They also include lack of child care and exercise partners,  and other social support factors, including negative family attitudes [10, 13]. By contrast, environmental factors are those which are outside of the mothers’ personal control and direct experience. These include access to public transport, access to recreational facilities, neighborhood safety and lack of an informative health system [10, 16]. The predominant effect of these barriers in restraining exercise participation is suggested in several studies [1, 8, 17].

Research needs to focus on the complex interplay of both personal and environmental barriers to PA in the lives of postpartum women. Results of such research can provide evidence for social support strategies in the form of exercise programs and changes to social and health policy. Such a strategic approach will enhance PA participation which is clearly a complex behavior that can be challenging to initiate and maintain [18]. Research has suggested that these determinants may be best described by incorporating a social-ecological model [19, 20]. Based on this framework, a thorough exploration of factors inhibiting or enabling PA participation is required [20]. Such a framework has been shown to be efficacious in enhancing PA behavior of latina women [21], and the incorporation of such a framework may also enhance PA behavior in women of childbearing years.

The majority of postpartum research has lacked the incorporation of a social-ecological framework to clearly classify PA determinants. It has focused predominantly on identifying barriers to postpartum PA and lacked a broad exploration of PA enablers (e.g. partner support, social support and mothers’ groups) [1, 22–24]. In addition, to identify PA determinants, studies have mostly trialed surveys [1, 23] or telephone counselling interventions [22, 25] and have lacked an autonomous qualitative component [1, 22–25]. For example, Cramp and Bray [1] in their survey study of 230 women investigated barriers to PA and examined exercise self-efficacy and barriers as a predictor of postpartum PA. Results mainly identified the role of personal barriers and self-efficacy as an important predictor of PA. Interestingly, the authors concluded that while self-efficacy was an important predictor of PA, it would be valuable to examine the role of environmental factors as well as a broader range of enablers in understanding PA behavior [1].

Fjeldsoe et al., (2010) delivered a 12 week exercise intervention via mobile telephone SMS on 80 postnatal women using two physical activity goal setting consultations with a focus on social support and self-efficacy as PA mediators. Findings suggested a significant positive effect on PA frequency, although no significant interaction effects were observed in PA duration [23]. Albright and colleagues in their study implemented a 12 months intervention on 311 postpartum women utilizing theoretically derived counseling techniques to enhance PA participation such as goal setting, problem solving and identification of barriers [22]. In their findings, the authors concluded that telephone counselling based on goal setting somewhat facilitated PA participation; however, findings lacked the investigation of other determinants that may enhance PA (e.g. partner support, social support and mothers’ groups) [22].

In addition to the interventional studies, Evenson et al.’s, study was the only study that integrated a qualitative approach to maternal beliefs, barriers, and enablers to PA [8]. The incorporated methodology restrained the findings (e.g. the designed questions were self-reported and were limited with only two open-ended questions). More importantly, these studies mostly have targeted women of specific ethnic backgrounds [1, 23], which inhibits a broader understanding of findings. Considering these existing limitations more research should be conducted about postpartum women’s attitudes to, and feelings about, both barriers and enablers to PA. The adoption of a social-ecological framework extends the understanding beyond purely personal factors. An in-depth qualitative approach allows women’s own voices to be acknowledged and gives insight into the particular contexts in which new mothers participate, or not, in exercise. Thus, the aim of this qualitative study was to provide an in-depth understanding of women’s beliefs about PA and exercise participation during the postpartum period by incorporating the social-ecological framework.

## Methods

This qualitative study was conducted with a subset of postpartum women who had previously completed the postpartum life style and physical activity survey which measured the facilitation of exercise participation, barriers and enablers, in childbearing years [2]. The research received ethics approval from two governing bodies (Curtin University and the Child and Adolescent Health Service, Western Australian Department of Health) and data collection commenced in March 2010 and was completed in July 2010.

A subset of 14 postpartum women (*N* = 14) six weeks to 12 months postpartum who had completed the survey study previously [2], and who had indicated an interest in continuing the study (which was an option given at the end of the survey) were subsequently invited to participate in interview sessions. Of the 150 women who completed the postpartum life style and physical activity survey [2], 30 women indicated they would be interested in being interviewed and of these we were able to contact and recruit 14 for the interview. Pertinent questions were based on the survey results and covered subjects related to: motherhood; life after giving birth; motivation to exercise; availability of social support such as mothers’ groups, community classes and mother’s exercise groups; and how mothers’ physical activity participation was either constrained or enabled following childbirth. Each participant signed an informed consent prior to her participation. All participants were interviewed in their own homes as this was more convenient due to child care responsibilities and time restrictions. Women were interviewed using a digitally recorded, face to face, semi-structured interview format. The interviews lasted for approximately 45 min. To determine the barrier and enablers to exercise participation, the interview questions were based on the social-ecological framework with an aim to clarify the interplay of personal, social, environmental and policy-related factors. For instance, the role of personal factors was determined by probing questions concerning task prioritizing after childbirth; the role of social determinants was explained by stipulating the role of partner support, social support and mothers’ groups. In addition, the role of environmental and policy related factors was identified based on the questions concerning the interaction between community health centers and local gyms in facilitating postpartum exercise classes.

The interview data were transcribed and thematic analysis was conducted through repeated reading of the transcripts, line by line analysis of the text and through repeated discussion between the authors. Initially to understand what was being stated by each of the participants, the first author read each of the transcripts line by line several times. After initial coding, codes were added and refined based on a second reading of the transcripts and a codebook was created. Based on the codebook, the second author independently coded the data. Discrepancies in coding were discussed between the coders until consensus was achieved. Subsequently an inductive approach was incorporated to assign a ‘description’ for each event and idea [26]. Significant sentences and phrases that contributed to a particular concepts were identified. Based on these descriptions preliminary ‘themes’ were identified.

The analysis identified general concepts related to postpartum life as well as specific issues related to the facilitation of exercise such as mother’s groups, community related classes, and barriers and enablers to physical activity participation. Finally, themes were refined and sub-themes were identified and concepts related to the social ecological framework were clarified and expanded within the themes.

### Trustworthiness

To maintain the credibility of the research upon the completion of the interviews and during data analysis the peer review process was incorporated continuously [27].

## Results

All of the participants were second time mothers, were between 18 to 43 years old and were 6 to 52 weeks postpartum. They were from a variety of ethnic backgrounds, but most were Australian (60 %) or British (30 %). Participants were all married; about 35 % had postgraduate qualifications and 25 % had done an undergraduate degree while 40 % of mothers had completed a college, vocational or secondary school qualification. In terms of employment, 53 % had been in a non- paid position (unpaid maternity leave); however 47 % had been in a full-time position which was followed by 27 % and 26 % respectively involved in part-time and casual positions. The highest annual household income was reported to be $120,000 per annum with the incomes of $75,000 to $119,000 being the second highest. In terms of exercise participation, only three participants out of 14 indicated that they were engaged in regular PA (e.g. walking, bike riding) on a daily basis; in addition pram walking was described as the activity most commonly and regularly used. The following results section involves three major themes followed by five sub-themes.

### Birth as a life altering event

During the interviews all of the women spoke about how hard it was to adjust to life after giving birth. Most women focused on the challenges of adjusting to their new lives and very few commented on the positive aspects of the experience. For example, some of the women expressed a sense of social isolation as they were ‘doing things by themselves’ and a few spoke of feeling ‘frustrated’ and ‘bored’ and struggled with the monotony of their new life. All spoke of feeling exhausted and some felt an upheaval in emotions ranging from sadness to extreme happiness which many participants explained as ‘hormonal imbalances’. The postpartum mood swings were generally viewed as short term.

Despite the difficulties women faced, most saw a way forward. For example, many of the women believed that the difficulties they experienced would be short lived and they would return to their normal life before long. Adjusting to their new lives also included reflecting upon and coming to terms with the event of childbirth. The women acknowledged that despite all of their preparation the birth experience was still unpredictable and for some uncontrollable but they generally believed they had managed well. Women spoke about changing expectations and how they were no longer thinking only of themselves. One of the women stated that ‘the highest priority is the baby’ (Participant (P13) and the other participant reported she needed to ‘feel in charge and in control of my baby’ (P8). However, not all women spoke this way and a number felt they deserved an opportunity to be involved in the kinds of activities they had previously enjoyed. One mother said: ‘It can’t be just about the baby’ (P5). For one mother keeping up her exercise routine was her main priority after having her baby (P4). The process of recovery from childbirth and adjustment to a new life was highly reliant upon the support of partners and family. For example, one woman said ‘with the help of my family I expect to get back to normal life soon’ (P1).

Overall, most of the women in the study were very dependent on their network of friends as well as their families to assist them to adjust to their new life. They saw their friends as a crucial component of their postpartum life.

### Postpartum barriers to physical activity following childbirth

The barriers to participating in PA in the postpartum period can be divided into personal and environmental factors, though both are often interconnected. Of the personal factors, lack of time was cited as one of the most common barriers.

#### Personal barriers

Very few women in the study made exercise a priority with the three highest priorities noted as looking after the baby and other children, taking care of husbands/partners and finally housework. With exercise made a low priority it is not surprising that women also lacked motivation to exercise, which was possibly also aggravated by the social isolation experienced by a number of women. Almost every mother stated that having support or ‘an extra hand’ would be useful in order to start exercising and to stay motivated to exercise. However, this was quite a challenge for many, with one woman stating she felt she always had ‘a child attached to her’ (P11). One participant complained ‘it’s really hard to go out anywhere and find anyone to exercise with’ (P7). One mother said, ‘you need the motivation and maybe if you were going with someone else you’d make more of an effort’ (P12).

#### Environmental barriers

Most of the women faced a number of environmental barriers, mainly related to access to appropriate and affordable exercise facilities. About half of the women did not know about available classes through the community and recreation centers or how they might access particular supports in their area. In addition, many mothers believed that there was insufficient information and support made available through the community centers for postnatal classes.

‘The only way I found out about it was through the health clinic and that was the exercises at B Park and if your clinic nurse didn’t tell you about it or you didn’t see the pamphlet you wouldn’t know. So maybe something that you actually do need when you register… maybe the government needs to tell women’ (P9).

Of those women who had sufficient information, most did not enroll in classes due to lack of time, the high cost of classes and the inconvenient locations. ‘Being on a low income’ or ‘expensive memberships’ were reasons mentioned by mothers for not using the classes and activities.

Most of the mothers agreed that they needed ‘the right exercise program’ in order to achieve maximum health after childbirth. All of participants mentioned the lack of a good quality program as a barrier to exercising. Some women expressed concern about the lack of a specific postnatal program, which would target pelvic floor dysfunction.

‘I need exercises that are simple and that you can do by yourself at home, exercises that would help with the pelvic floor because that is really important because I didn’t realise how important it was until I had this baby because I was so tender I couldn’t feel that part of my body’ (P6).

A number of women had participated in postnatal swimming classes but these were only for a set period of time and once they stopped women tended to stop exercising altogether. Therefore, due to the inefficiency of the available exercise programs women tended to avoid exercise classes and as a result over a specific period of time they were less confident in participating in physical activity or spoke about having a poor body image.

### Postpartum exercise enablers following childbirth

#### Partner support

Most of the study participants described having partner support as ‘the most reliable source of support’. They believed the presence of their partners was vital to enable them to exercise. Some mothers relied on their partner to the extent that they assigned exact times when they could expect to receive the needed support; for example one participant mentioned ‘I like to have the support on the weekend or late afternoon’ (P13). Sometimes mothers exercised with their partners; for instance one mother said, ‘I would rather go out walking with my husband and baby with the pram instead of a mothers’ group’ (P2). In addition, some mothers stated that having a physically active partner helped them to participate more in exercise as the partner encouraged them and understood the importance of them having their own exercise regimen. One participant used the term ‘planned walking with my partner’ (P4), indicating the importance of scheduling exercise into her family routine. It is important to note that sometimes partners could not help, even if they were supportive. For example, a number of mothers noted how breastfeeding was a time consuming task only they could undertake, or the baby would ‘not take the bottle from someone else’ or would ‘not stay with anyone else’.

#### Social support

Social networks, like mothers’ groups and postnatal classes, were considered highly important for most of the mothers, both to share their experience and to create opportunities to be physically active. The role of the mothers’ group that many of the women attended was primarily to provide postpartum education and communication. A number of the women said they felt ‘fantastic’ after participating in various groups and community workshops and most women said it was important to meet others in a ‘similar situation’. The existence of the mother’s group was described as ‘great’ by most of the mothers. Mothers believed they could share the same stories and experiences in regards to motherhood and the responsibilities of being a mother.

‘We’ve all got a lot in common and I think that’s probably why we’ve stayed such a good group and so close’ (P2).

In addition, half of the mothers pointed out how having the same age babies increased the likelihood of maintaining one’s relationship with the mother’s group.

‘I guess the biggest thing we have in common is that we have babies the same age, and that’s kind of the biggest factor in your life at the moment… So that’s probably what we do and I suppose we have other things in common but you kind of discover those as you are talking. I suppose it’s just nice to go to a place where you can talk to people who are having similar problems or experiences so that’s good’ (P4).

Interestingly, most of the mothers frequently described how the mother’s group could provide a supportive atmosphere to participate in more structured exercise sessions such as pram walking.

‘There are 15 of us that have stayed in the mothers’ group and we continue to meet at the community center once a week and it’s been a really good source of support. Just to chat with other mums every week about what they’re going through and just to feel that it’s quite normal. On occasion we’ve met at a park. So that’s why walking with the pram is such a good option because you just take the baby with you and the baby will sleep’ (P5).

Although most of the mothers belonged to a local network of other new mothers, a few chose not to join the groups due to ‘age restrictions’, ‘having more in common with a person not a group’, ‘being too busy’ or because they chose to rely on their own network of friends.

#### Postnatal exercise classes

Prenatal and postnatal classes such as swimming sessions, on the other hand, were discussed as a suitable and a structured atmosphere to encourage exercise participation during transition to motherhood and in the early months of the postpartum period. Some of the mothers described it as very affordable and a place to meet other mothers and share their motherhood journey.

‘I went to postnatal swimming and I’d do that once a week, so that was 20 min with the baby in the water and then it was about three quarters of an hour for the mother to do exercises so that was good and that lasted ‘till she was about 5 months. And I also did, once a week, I went to the B Hospital for exercises. I was a bit upset about that it sort of ends at like 6 months and there was nothing after that… I think the problem is that there’s nothing after 6 months it’s all about just after the baby but nothing long term, you’ve got to pay for it’ (P 1).

The mothers tended to get involved in postnatal classes mostly through the hospital where they gave birth and for a limited period. The motivating factors to attend these classes included having free access to the classes, their baby’s participation with them in the class, or having someone to look after their babies while they were exercising.

‘I’ve been to the hospital where Sam was born. They have a mother’s group there which is really good and that was really helpful, lots of mums with lots of different ideas and we also went to the hospital postnatal swimming classes that are mostly for mums but the bubs do a little bit of splashing in the water when they’re really little so that was really good too’ (P10).

Identifying achievable goals and setting a standard achievable program were the key elements in enabling women to exercise [28]. Half of the mothers believed they had learned more about the variety of exercises available. The ‘exercise modification’, ‘how it targeted health’, and learning about ‘muscles, strength and endurance’ were mentioned as the best reasons to attend exercise classes.

One mother commented that ‘exercise is a stress reliever, and makes you feel energetic’ (P5). One participant stated she had a ‘positive feeling’ after exercise (P3) and yet one mother said ‘feeling mentally better’ (P9) is what encouraged her to exercise.

Despite the benefits of such group sessions, there were some limitations, such as being short in duration in the postpartum period and a lack of individual instruction. Due to the mentioned restrictions in regard to the community classes, some of the mothers expressed the desire to join a mother’s exercise club. About half of the mothers indicated that such clubs would benefit from instructors that specialized in postpartum physical health and facilities to accommodate the presence of babies while the mothers exercised or participated in exercise sessions with babies. In addition the mothers noted that having the ability to breastfeed their baby at an exercise club could be useful in overcoming barriers to exercise.

‘So if you wanted to do like a class, like a public group fitness class somewhere then you need that class to be at a time when your baby’s not going to need feeding, so feeding I think is a hurdle. It would be really good, to have an exercise club like mother’s group where you all go and meet up with them and just sit and chat and talk about things. If the same thing existed like a mother’s exercise club where you would go and all the new mums would go and once a week or once a fortnight and you’d all exercise together. So if something like that was there then I think that would really be really good for everybody’ (P 2).

## Discussion

The aim of this qualitative study was to provide an in-depth understanding of women’s beliefs about PA and exercise participation during the postpartum period. The analysis used the social-ecological framework to help understand individual, interpersonal, community and organizational factors that both created barriers to, and helped encourage, the women’s PA and exercise participation. Considering the significance of the social-ecological model in improving exercise participation [20], this study has highlighted its importance by focusing, not just on personal and interpersonal factors, but simultaneously considering how community and organizational factors can both hinder and encourage exercise participation. The model was incorporated by determining personal factors such as mothers’ knowledge and attitude towards exercise (e.g. task prioritizing); in addition interpersonal determinants were described by investigating the role of partner support, social support and mothers’ groups. The interaction between different organizations (e.g. hospital, community health centers, and local gyms) signifies the importance of the community as a determinant of PA. Also this study depicts the interaction between organizational and policy determinants by emphasizing the need to encourage hospitals, community health centers, exercise providers and policy makers about the importance of postpartum health and specifically the role of exercise in improving new mothers’ physical and psychological health and well-being.

The strength of this study was its design, and to our knowledge this is the first face-to-face qualitative study evaluating postpartum women’s perspectives regarding both barriers and enablers to exercise participation. Previous studies were deficient in thoroughly investigating exercise enablers in postpartum women [1, 8, 22], were mainly based on problem solving barriers to PA [23, 25] and lacked a stand-alone qualitative component [24]. In addition, our study covered a broader range of themes and sub-themes in alignment with the social-ecological framework.

In detail, our findings suggest that Western Australian postpartum women face similar challenges to motherhood and barriers to PA when compared with childbearing women elsewhere [9]. For instance, the barriers reported by the study participants were consistent with barriers reported in other studies (e.g. being tired and lack of time) [22, 29]. In addition, both pregnancy and postpartum life decreased mothers‘activity level generally [13, 30]. The obstacles to postpartum PA were many and it may not seem surprising that the study participants considered lack of energy, social isolation, and lack of access to a feasible exercise program as major barriers to exercise and other forms of physical activity.

Our study provides more important insights; it illustrated that mothers were aware of the general benefits of exercise; however, when exercise was left for them to organize, they did not prioritise it. This was due to often feeling overwhelmed by motherhood responsibilities, lack of energy, feeling exhausted, time restrictions and generally considering exercise a low priority. As found in other studies, the new mothers considered childbirth and parenting as the biggest responsibility they would ever face [31]. As a result, our findings suggest the importance of establishing time management skills and maintaining a routinely pre-organised activity to overcome these barriers. Not only were the study participants exhausted from their new parenting responsibilities, it appeared that they were also required to find information about where and how they could exercise and what would be the available resources available to them.

Similarly Evenson and colleagues stated that lack of professional advice regarding a feasible exercise program was a barrier to exercise participation [8]. These findings suggest that appropriate authorities such as health professionals and health care practitioners need to help postpartum women to participate in exercise, particularly once the early postpartum support has stopped. A number of the women mentioned that once they completed some of the mother and baby exercise programs, there was nothing available to them. An argument can be made that increased funds are required to provide ongoing exercise programs suitable for mothers.

A number of mothers who participated in this study were worried about being socially isolated and not having someone to look after their baby and this impacted greatly upon their ability to become involved in exercise programs. Our findings are consistent with other recent studies stating childcare as a barrier to exercise participation [8, 32]. Therefore, there may be a good argument to develop broad based classes for postpartum women with respect to the importance of social support to lessen the effect of isolation as a barrier.

The current study confirmed the positive effect of social support in enabling exercise and the findings are in accordance with others [23, 33, 34]. Similarly, the role of partner support is of great importance in facilitating exercise participation and this has been confirmed generally in other quantitative studies [6, 17, 30, 32, 35]. In addition, the women in this study relied heavily upon their partners as well as their friends who they valued greatly. Despite this, a number of mothers reported difficulty in finding someone to exercise with. Therefore, social networks of any kind, for example, mothers’ exercise club and mothers’ group can be a great support for postpartum women wishing to exercise. While not all the participants valued exercising with other mothers, a number of strategies are required and individualized programs should be made available to new mothers based upon their needs and preferences.

Previous studies have conducted their exercise trials without prior investigation of barriers and enablers to exercise [1, 22–25]. Therefore, the findings from our study are of importance in tailoring future exercise interventions for postpartum women as it seems that individualized programs are likely to be more acceptable to postpartum women. Overall, this study provides in-depth information to create more successful strategies to enhance regular PA participation in postpartum women.

### Strengths and limitations

The findings reported here have been illustrated through an in-depth qualitative study which follows a previously published quantitative study that analyzed the findings of a survey study [2]. Indeed one of the strengths of this study was the fact that interview questions were developed partially from a previous survey study [2]. A limitation of the study was that the women’s responses might change based on the duration of time since the birth of their baby and this requires a longitudinal methodology to address this issue. In addition, a bigger sample size with a clinical condition such as a postnatal depression would extend the study further and provide greater confidence in the results.

In terms of methodological limitations, as mentioned previously, participants from the earlier survey study (*n* = 150 postpartum women) agreed to be part of the interview. The initial response rate was 20 % as specified as an option given at the end of the survey. However, when the research team contacted the potential participants the response rate was dropped to 10 % as only 14 women were available. This resulted in a small sample size which was not purposive. Due to the small numbers full data saturation may not have been achieved. However, there were several recurring concerns and suggestions for change which are evident in the study themes and sub-themes regarding barriers and enablers. Despite the small numbers the in-depth nature of the approach allowed the research team to get a clearer picture of the issues involved for this group of women.

Another limitation was that the women were not all similar in postpartum age; for example some were closer to the birth than others. However, this allowed the research team to identify a broader range of barriers and enablers across the postpartum period for the women involved. Interestingly, most of the group had partners and were well-educated. Therefore, this study only captures the views of a particular demographic. Women who do not have partners or who have a lower standard of education may experience a greater level of social isolation or inability to participate in paid exercise classes.

## Conclusion

Evidence from this study has relevance to public health and policy advisors as well as health practitioners who advise women in the postpartum period. A range of strategies are needed to support postpartum women to engage in exercise as there is no ‘one size fits all’ approach. Generic exercise programs may not be a solution as this study demonstrated that mothers have different needs, priorities and goals. While some mothers would exercise in groups given the appropriate supports, others were not interested in group participation and requested individualized exercise programs. No matter what exercise program is chosen, it must be both achievable and accessible. Mothers need information about childcare support, the availability of postpartum classes and, if appropriate, how they can tailor a home-based program to meet their own needs. The study findings demonstrate that further investigation through practical intervention is warranted. More research needs to be done to explore postpartum lifestyles and how targeted exercise interventions affect the health and well-being of postpartum women.

### Abbreviations

PA, physical activity.

## Additional files

Additional file 1: Interview Guide - contains interview questions. (DOCX 13 kb) Additional file 2: Consolidated criteria for reporting qualitative studies (COREQ): 32-item checklist. (DOCX 17 kb)
