# Supplementary material for: Perceived barriers and enablers of physical activity in postpartum women: a qualitative approach
Source: BMC Pregnancy Childbirth. 2016 Jun 2;16:131. doi: 10.1186/s12884-016-0908-x (PMC4890285; doi:10.1186/s12884-016-0908-x)
Supplement: Additional file 1: — Interview Guide - contains interview questions. (DOCX 13 kb) [file 12884_2016_908_MOESM1_ESM.docx]

## Interview Guide

1- How have you been feeling since the birth of your baby?

2- What are the priorities for a woman after child birth?

3- Would you consider yourself as someone who is doing moderate to vigorous activity?

4- What sort of activity do you do? On a daily basis/ on a weekly basis?

5- What sort of exercise do you enjoy?

6- Have you been able to do this exercise recently?

7- If no – why? If yes- why?

8- What do you think generally are the barriers to women exercising after child birth?

9- What do you think makes a woman start exercising after child birth?

10- What kinds of support do you think you need in order to exercise?

11- What do you think your partner needs to do to help you?

12- How do you think exercise could help you?

13- What type of an exercise program would be the easier to adhere to?
